# A Case of an IgG4-Related Disease Mimicking Malignancy and Resolving With Steroids

**DOI:** 10.7759/cureus.9476

**Published:** 2020-07-30

**Authors:** Varun Samji, Tarek Haykal, Rizwan Danish, Ghassan Bachuwa

**Affiliations:** 1 Internal Medicine, Hurley Medical Center, Flint, USA; 2 Internal Medicine, Hurley Medical Center, Michigan State University, Flint, USA; 3 Hematology and Oncology, Hurley Medical Center, Michigan State University, Flint, USA

**Keywords:** igg4-related disease, pseudo tumor, igg4 related pseudotumor, inflammatory pseudotumor

## Abstract

A 77-year-old African American female was referred to oncology for evaluation of an adrenal fossa mass detected on computed tomography scan of the abdomen and pelvis (CT-scan A/P) that was ordered as a work-up for painless hematuria. Further evaluation by positron emission tomography (PET) scan showed hypermetabolic masses in the left suprarenal and right iliac region. The biopsy of the right iliac mass was consistent with IgG4-related disease (IgG4RD). It was supported by an elevated serum IgG4 level. She was treated with prednisone with a good response.

## Introduction

IgG4-related disease (IgG4RD) is a recently recognized systemic disease. The disease spectrum shares similar characteristics of tumour-like swelling of the involved organ, IgG4-positive plasma cell infiltrate, storiform fibrosis, elevated serum IgG4 levels in most of the cases and a good response to steroids [1].

IgG4RD has been described in multiple organ systems [1]. IgG4RD presenting as pseudotumor of orbit, brain, liver, pituitary, and kidneys has been described [2-4]. In case reports of IgG4RD presenting as pseudotumor of kidneys, patients underwent partial nephrectomy as the mass was thought to be malignant [5]. This is a case of IgG4RD presenting as tumour-like suprarenal and pelvic mass without systemic symptoms. The patient is treated with steroids. In patients who are being evaluated for tumour-like masses, IgG4RD should be considered as one of the differential diagnoses to try and avoid surgical interventions.

## Case presentation

A 77-year-old African American female presented to her physician with a main complaint of painless hematuria of one-month duration. She denied flank pain, back pain, groin pain, nausea, vomiting, fever and chills. She also denied any weight loss. Her past medical history included essential hypertension, mild intermittent asthma, osteoarthritis and peripheral vascular disease. Her past surgical history included hysterectomy. Significant family history included pancreatic cancer in mother and lung cancer in father. She had a history of smoking cigarettes for 15 pack years, however, she quit two years earlier. She reported being a social drinker. The patient had no history of allergies. Her recent Pap smear, mammography and colonoscopy were up-to-date and unremarkable. Review of systems was positive for fatigue. Physical examination was unremarkable.

**Figure 1:**
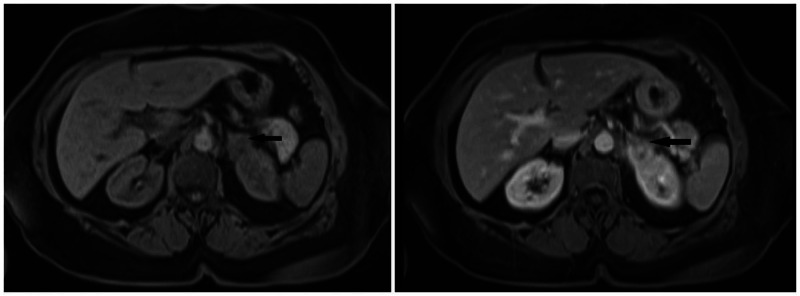
MRI of abdomen W/O and W/contrast showing left suprarenal mass inseparable from the upper pole of the left kidney and left adrenal gland (arrow) with T1 and T2 slightly hypointense signal compared to renal cortex, restricted diffusion and postcontrast enhancement.

**Figure 2:**
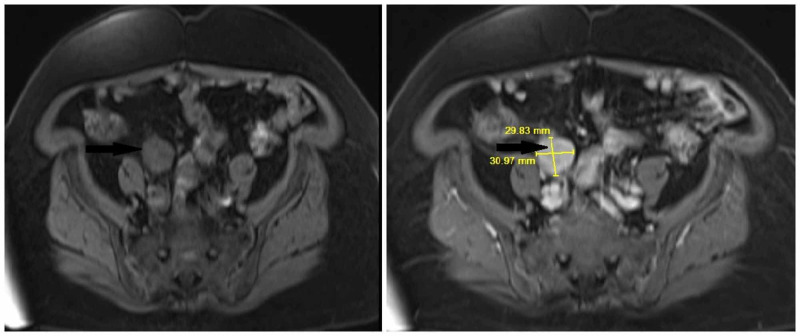
MRI of abdomen W/O and W/contrast showing similar appearing lesion in right pelvis just anterior to the common iliac arteries measuring 3.1 x 3.0 cm (arrow).

The patient’s complete blood count showed a white cell count of 9300 cells/dl with normal differentials. Hemoglobin was normal at 12.6. She had normal liver enzymes. Renal function was normal with serum creatinine of 0.9. Urine dipstick was positive for blood and trace leukocyte esterase, but negative for glucose, bilirubin, and protein. A CT-scan of abdomen and pelvis with intravenous contrast was performed, which showed a 3.3 x 2.3 cm left adrenal gland fossa mass with superior perinephric nodular fat stranding and a 3.2 x 3.2 cm retroperitoneal mass anterior to the right iliac artery bifurcation. CT scan also showed a 2.1-cm partial staghorn calculus in the left kidney. A cystoscopy was done, which was negative for a bladder mass. Magnetic resonance imaging (MRI) of the abdomen showed a soft tissue mass in the left adrenal gland fossa inseparable from the upper pole of the left kidney and left adrenal gland with T1 and T2 slightly hypointense signal compared to renal cortex, restricted diffusion and post contrast enhancement (Figure 1). A similar-appearing lesion in the right pelvis just anterior to the common iliac arteries measuring 3.1 x 3.0 cm was seen (Figure 2). A positron emission tomography (PET) scan was done, which showed hypermetabolic masses in the left suprarenal region and right iliac region with no other fluorodeoxyglucose (FDG) avid disease.

**Figure 3:**
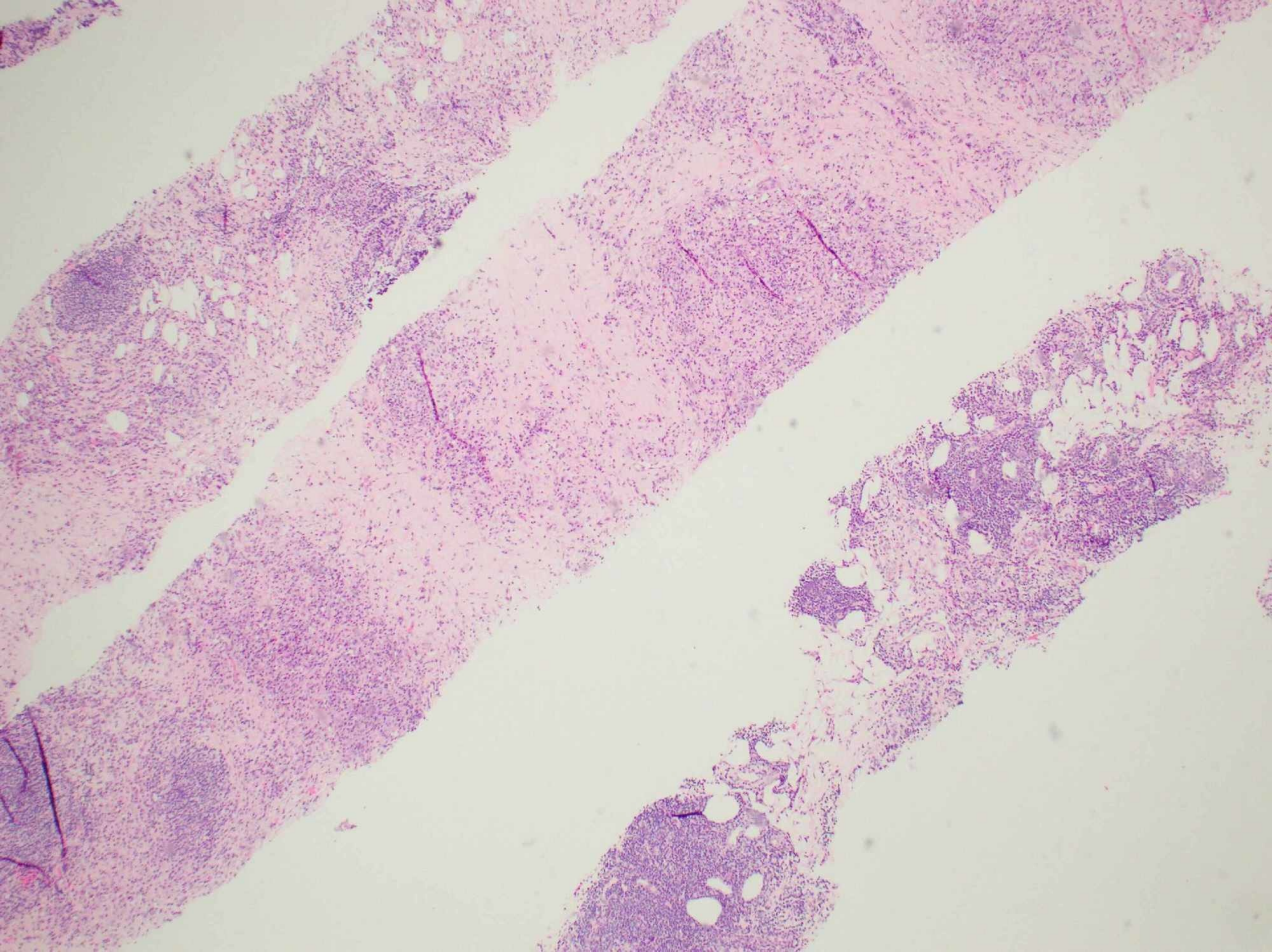
Hematoxylin and eosin stain sections of the core biopsy showing alternate areas of fibrosis and inflammation.

**Figure 4:**
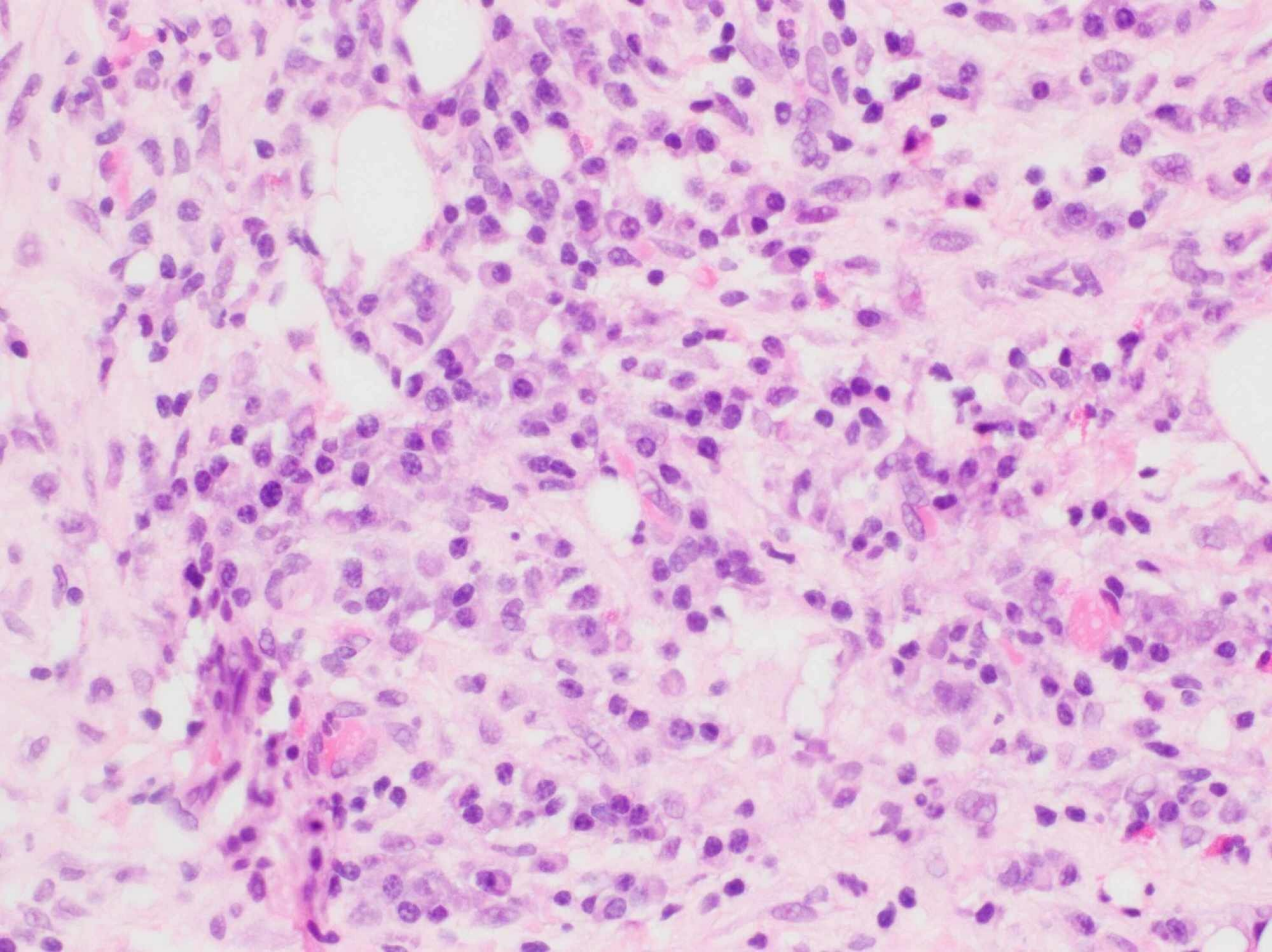
Hematoxylin and eosin stain under 40X showing increased plasma cells.

**Figure 5:**
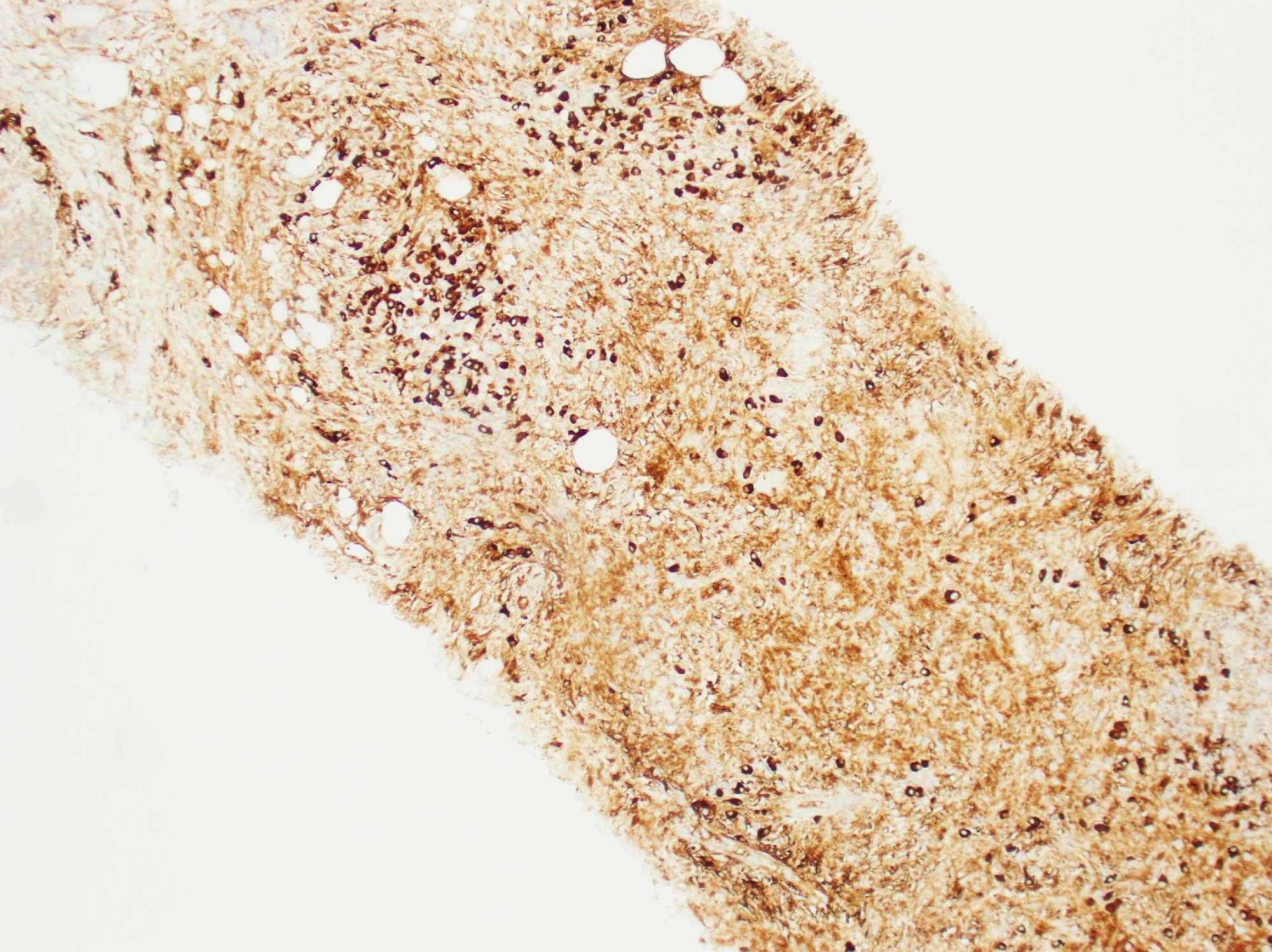
IgG4 staining under 10X showing more than 40% staining of IgG4-positive plasma cells.

A CT-guided biopsy of the right iliac mass was performed. Hematoxylin and eosin stain sections of the core biopsy showed fibro adipose tissue with extensive fibrosis, scattered lymphoid follicles and areas of plasma cell-rich lymphoplasmacytic infiltrates (Figures 3, 4). By immunohistochemistry, the IgG4 to IgG ratio was greater than 0.4 and there were more than 10 IgG4-positive plasma cells per high power field (Figure 5). There was no evidence of lymphoproliferative disease. The patient was tested retrospectively for IgG4 level, which was elevated to 140.7 mg/dl (normal range: 4-86 mg/dl).

The differential diagnosis included renal cell carcinoma, lymphoma, sarcoma and carcinoid tumour. Given the suprarenal mass and retroperitoneal lymphadenopathy, cancer was highly suspected. The biopsy of the iliac mass was performed, which showed alternating areas of inflammation and fibrosis. CD 134 stain showed increased plasma cells which were positive for IgG4 stain. IgG4 stain showed more than 40% plasma cells. The diagnosis of IgG4RD disease was made based on tissue biopsy.

**Figure 6:**
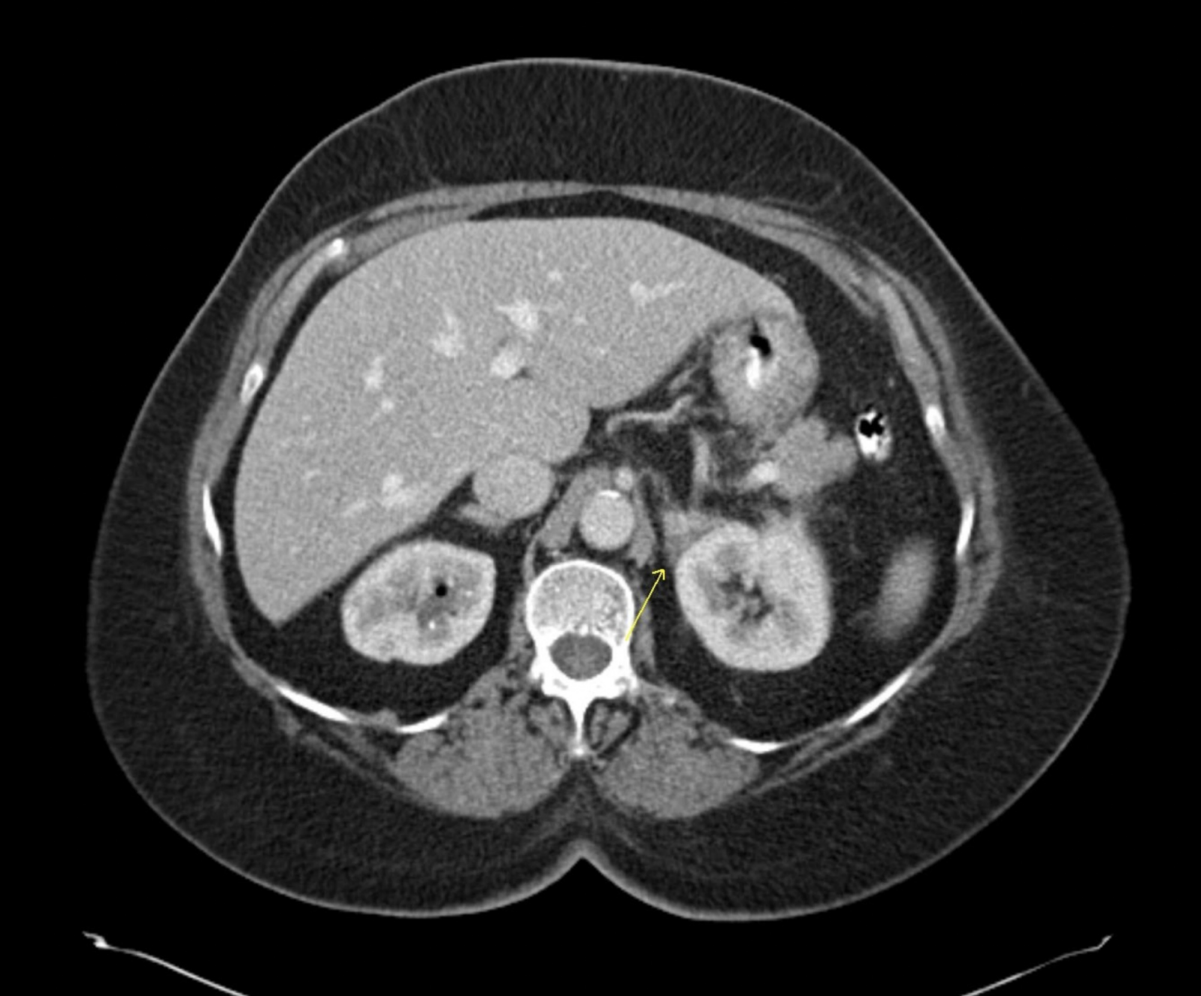
CT of the abdomen W/contrast showing decrease in soft tissue thickening of the suprarenal mass (arrow).

**Figure 7:**
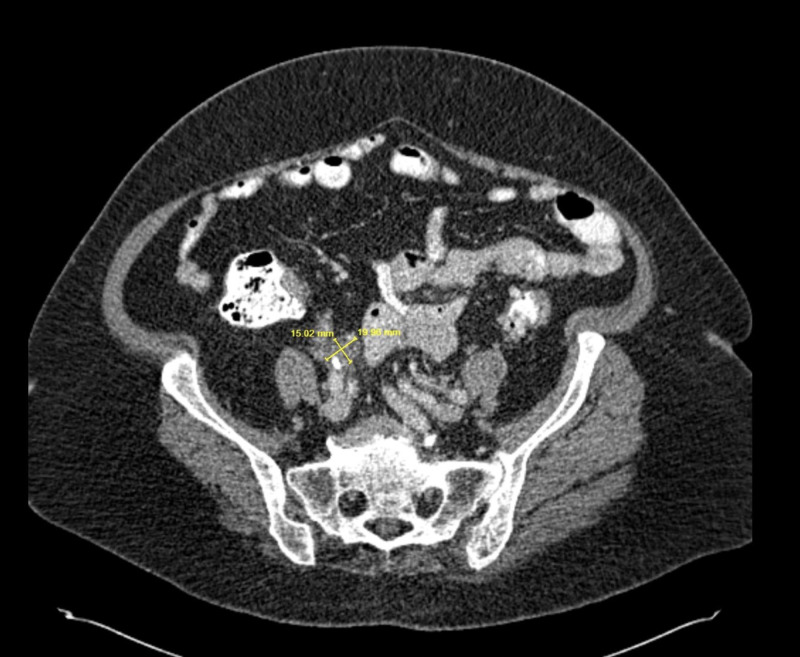
CT of the abdomen W/contrast showing decrease in soft tissue mass just proximal to the iliac bifurcation along the right common iliac artery.

A diagnosis of IgG4RD was made and the patient was started on prednisone 20 mg daily. Approximately one month after initiating steroid therapy, the patient had a follow-up CT scan, which showed interval decrease in size of the left suprarenal soft tissue thickening, soft tissue nodularity in the left perirenal space and retroperitoneum soft tissue mass at the right iliac artery bifurcation (Figures 6, 7).

## Discussion

In 2001, high serum IgG4 concentration was linked to a unique form of sclerosing pancreatitis, which is now known as type 1 autoimmune pancreatitis (AIP) [6]. Kamisawa et al. demonstrated IgG4-positive plasma cells infiltrate systemically in patients with AIP and proposed a new clinicopathologic entity called “IgG4-related systemic autoimmune disease” [7]. A number of medical conditions such as Mikulicz’s syndrome, Kuttner’s tumor, and Riedel’s thyroiditis which were regarded as conditions confined to single organs are now viewed under IgG4RD [1].

IgG4RD has been described in multiple organ systems [1]. The disease presents subacutely. It can involve one major organ and affect other organ systems over years or multiple organs can be involved on presentation. Most of the time it is detected incidentally through imaging or through pathology specimens [1]. Clinical features follow the organ system affected. It can present as swelling of affected organs, organ dysfunction such as pancreatitis, hypothyroidism, hypopituitarism, obstructive jaundice, deterioration of kidney function, or retroperitoneal fibrosis causing obstructive uropathy [1]. It can also present as isolated tumour-like swelling of the involved organ. The pancreas, hepatobiliary system, kidneys, and retroperitoneum are the common sites of intraabdominal IgG4RD [8]. IgG4RD presenting as pseudotumor without any other signs or symptoms is a diagnostic challenge as it is often confused with malignancy [5].

Epidemiology of IgG4RD is poorly described. In Japan, the prevalence of patients with autoimmune pancreatitis was estimated to be 0.82 per 100,000 and was predominantly seen in men over 45 years of age [9]. In a cross-sectional study of 114 patients with IgG4RD, researchers divided patients into five groups (head and neck, thoracic, hepatic and pancreatobiliary, retroperitoneal and systemic) based on the location of lesions [10]. The ages in each group were similar with means ranging from 59 to 68. All groups except the one with head and neck involvement were predominantly men. The group with only head and neck disease was divided more equally with 48% of the patients being men [10].

Histopathology is critical in diagnosis and also differentiating from malignancy. The major histological features associated with IgG4RD include a dense lymphoplasmacytic inflammatory infiltrate, characteristic storiform pattern of fibrosis and obliterative vasculitis [1,11]. The infiltrate predominately consists of IgG4-positive (+) plasma cells and mild to moderate eosinophils [11]. Immunohistochemistry is required for confirmation of diagnosis [1]. Immunohistochemical staining aids in quantifying IgG4 cells; it is usually measured in IgG4+ cells per high power field or IgG4 to IgG ratio [11]. Imaging helps in detecting the extent of disease and response to treatment, but does not differentiate IgG4RD and cancer [1,8]. The involved organ shows focal or diffuse enlargement; MRI usually shows late enhancement and restricted diffusion. PET scan shows increased uptake by the involved gland. Resolution of findings is seen in response to treatment [8].

The Mayo Clinic Criteria and Japanese Society of Nephrology are two sets of proposed diagnostic criteria [12,13]. To diagnose IgG4RD, > 10 IgG4+ plasma cells/HPF and/or an IgG4+/IgG ratio > 40% on histology plus elevated serum IgG4 or IgG levels or characteristic IgG4RD features at other sites or imaging features of IgG4RD described above are required for diagnosis [13].

Treatment of IgG4RD in asymptomatic individuals is still controversial [14]. The mainstay of treatment of IgG4RD is corticosteroids [15]. The treatment protocols for IgG4RD are still evolving. Use of alternative agents such as rituximab is still under investigation [16]. IgG4RD is treated with prednisone 0.6 mg/kg/day for 2-4 weeks. The dose is gradually tapered by 5 mg every 1-2 weeks depending on response to treatment to a maintenance dose of 2.5 to 5 mg/day over a period of 2-3 months [17]. Maintenance therapy is required in multiorgan involvement, elevated IgG4 levels and relapse. Rituximab can be used while treating steroid-resistant or intolerant cases [18].

In our case, the patient had no signs or symptoms of IgG4RD or any other systemic illness. A suprarenal mass and an iliac mass at the right iliac artery bifurcation with similar signal intensity were detected on imaging. IgG4RD was confirmed by biopsy of the iliac mass. Only three cases of IgG4RD presenting as an isolated renal mass without systemic signs of disease have been reported. Two of the three cases underwent surgical intervention [5]. Histopathology, IgG4+cell counts and IgG4 to IgG cell ratio will help in confirming the diagnosis of pseudotumor [19]. Although close observation was one of the approaches, with a suprarenal mass and a retroperitoneal mass close to a major blood vessel, the decision was made to treat the patient with oral glucocorticoids. CT-scan after treatment showed good response to treatment.

## Conclusions

IgG4RD is a multisystem disease which is most commonly described in the pancreas and can involve virtually any organ system. Isolated presentation of IgG4RD presenting as pseudotumor is rare and is always worked up as malignancy. It requires a very high index of suspicion; imaging features and serum IgG4 or IgG levels may help in pointing towards IgG4RD. Early diagnosis is essential since it is a treatable illness and if untreated it is debilitating. The treatment for IgG4RD is still evolving; the current recommendation is to treat patients with corticosteroids. The second line of treatment is rituximab if steroids are not tolerated, the case is steroid-resistant or in relapsed disease.
